# Reflex Symptoms Consequent upon Ptosis of Kidney.—Operation

**Published:** 1905-01

**Authors:** L. G. Hanley

**Affiliations:** Buffalo, N. Y., Buffalo Hospital Sisters of Charity


### Reflex Symptoms Consequent Upon Ptosis of Kidney.—Operation.

By L. G. HANLEY, M. D., Buffalo, N. Y.,
Buffalo Hospital Sisters of Charity.

MRS. G., age, 46; family history—mother and father alive and well; two sisters died when children; one brother dead, cause, rheumatism; five other members of family alive and well. Personal history: patient had measles and whooping-cough when a child; had malarial fever about eighteen years ago; menstrual periods, normal in character until five years ago, when she suffered from dysmenorrhea and also profuse menorrhagia, which has continued for five years; never pregnant; for about sixteen years has complained of periodical gastralgia, at which times she suffered from distention of gas in the bowels, pyrosis, and considerable pain in epigastric region. All these symptoms were somewhat relieved by vomiting, which was induced by herself.

She has been subject to attacks of dizziness resembling vertigo, with some suppression of urine at these times. The most severe attack of gastralgia occurred five years ago, when she was confined to her bed for several months. Several physicians of repute made a diagnosis of gastric ulcer at this time. She became greatly emaciated until she was reduced to 90 pounds. A tumor in the abdomen was discovered, which proved to be a movable kidney of extreme degree. The patient has had for the last few years, at several intervals, symptoms similar to those described; she has worn a bandage and says, "she always found great relief when her kidney came down by pushing it back into place." She could, when she so desired, push her kidney to the lower part of the abdomen and back again.

Present illness: November, 1904, she was seized with severe pain in her back and abdomen, vomiting, frequent and scanty urination, and experienced great distress in trying to replace kidney. Examination of the stomach contents showed normal digestion and absence of blood. Vomiting persisted for one week, resisting all treatment. These symptoms, it was thought, might be relieved by surgical intervention, therefore operation was advised and the patient removed to hospital. Catheterisation of either kidney showed the urine to be normal in quality from each ureter; also further examination revealed the presence of subperitoneal fibroids with retroverted uterus.

On opening the abdomen two myomata of the size of small oranges were removed and the uterus was anchored to anterior abdominal wall, Hanley's method of ventral fixation being employed. Another incision was made through the quadratus lumborum and a kidney measuring 6½ inches in length, 3½ inches in width, and 2 inches in thickness was drawn into the opening. On account of the size of the kidney, the emaciated condition of patient and the small amount of perinephritic fat, a nephrectomy instead of a nephropexy was performed.

From November 8 to December 14, she did not vomit; she takes ordinary diet and has gained in weight; whereas, formerly the stomach would retain no food and she was sustained by nutrient enemata. She suffers no pain and feels perfectly well. It seems apparent that her condition was due to extreme ptosis of the kidney.

---

INTESTINAL COLIC AND PERITONITIS.—A very bad intestinal colic may simulate an attack of peritonitis. In the first you can move the abdominal wall over the intestinal mass, whereas in the second the wall is rigid.—*Practical Medicine,* Delhi, India.

